# A Quantitative Method for Intraoperative Evaluation of Distal Fibular Malrotation

**DOI:** 10.3389/fsurg.2022.887004

**Published:** 2022-05-04

**Authors:** Hui Huang, Zihua Li, Fajiao Xiao, Jiang Xia, Bing Li, Tao Yu, Youguang Zhao, Haichao Zhou, Wenbao He, Zhendong Li, Yunfeng Yang

**Affiliations:** ^1^ Department of Orthopedics, Shanghai Tongji Hospital, School of Medicine, Tongji University, Shanghai, China; ^2^ Department of Orthopedics, Shanghai Tenth People’s Hospital, School of Medicine, Tongji University, Shanghai, China

**Keywords:** three-dimensional reconstruction, ankle fractures, distal fibula, rotational malreduction, intraoperative fluoroscopy

## Abstract

**Background:**

Due to the low sensitivity of commonly used radiographic parameters for the evaluation of rotational malreduction of the distal fibula under intraoperative fluoroscopy, a quantitative method is needed to make up for this defect.

**Methods:**

A total of 96 sets of computed tomography images of normal ankles were imported into MIMICS to reconstruct 3D models. The fibula models were rotated along the longitudinal axis from 30 degrees of external rotation to 30 degrees of internal rotation. Virtual X-ray function in MIMICS was used to obtain radiographic images in mortise view. A line was drawn through the tip of the medial malleolus and parallel to the distal tibial plafond, the distances from the medial edge of the fibula to the lateral malleolar fossa cortex and from the medial edge of the fibula to the lateral edge of the fibula were measured on this line, and the ratio of them was calculated and marked as ratio *α*.

**Results:**

The mean ratio *α* for normal ankles was 0.49 ± 0.06, while the 95% confidence interval was 0.48–0.50. The ratio *α* decreased when the fibula was externally rotated and increased when the fibula was internally rotated. The effects of different genders or different types on each group of data were compared, and the *p* values were all greater than 0.05.

**Conclusions:**

This is a new method to quantitatively evaluate rotational malreduction of the distal fibula during operation. The ratio *α* can correspond to the rotation angle of the fibula. The larger the ratio *α*, the more the internal rotation of the fibula. Contrarily, the smaller the ratio α, the more the external rotation of the fibula. Making the ratio *α* close to 0.5 may be an intuitive approach that can be used intraoperatively.

## Introduction

Malreduction of the distal fibula is usually related to the ankle fractures, and the types of fracture are mostly classified as Weber type C. Rotational malreduction of the fibula will change the contact pressure in tibiotalar (1), talofibular, and tibiofibular articulations (2), and clinical studies have also demonstrated that malreduction of the fibular often predicts poor functional outcomes (3–5). Postoperative computed tomography (CT) images showed that up to 52% of ankle fractures had malreduction of distal fibula (3, 5–7), including shortening deformity, tilting in the sagittal and coronal planes, and axial rotation. Among them, malrotation is most difficult to detect intraoperatively (8, 9). Most surgeons use mortise and anteroposterior radiographs and utilize various radiographic parameters, such as medial clear space, tibiofibular clear space (TFCs), and tibiofibular overlap, to assess the accuracy of reduction (10–12). However, the sensitivity to detect malrotation and lateral displacement of the distal fibula is far from satisfactory (5, 13–15). In contrast, intraoperative CT is more effective (3, 16, 17), but it is inconvenient and significantly increases radiation exposure for both the patients and the surgeons.

It is necessary to find a measurement that can accurately evaluate the rotational malreduction of the distal fibula under intraoperative fluoroscopy. Marmor et al. (18) and Chang et al. (19) proposed to observe the morphology of the distal fibula and the location of the lateral malleolar fossa cortex in mortise radiographic views to detect whether the fibula has rotational malreduction. However, due to the inability to measure quantitatively, these indicators can only be roughly assessed, and the reference range to define rotational malreduction of the distal fibula is yet still lacking.

The purpose of this paper was to use MIMICS (version 22.0, Materialise NV Technologielaan, Leuven, Belgium) to reconstruct 3D models of normal ankles and rotate the fibular models to simulate fibular malrotations. The vrtual X-ray function in MIMICS was used to obtain radiographic images in mortise view to explore whether there are better parameters to quantitatively evaluate the rotational malreduction of the distal fibular.

## Material and Methods

This study retrospectively analyzed 120 normal-ankle CT images of outpatients from January 2018 to July 2021. All outpatients were scanned in a non-weight-bearing position.

The inclusion criteria of outpatients were age ≥18 years and having clear CT images. The exclusion criteria were a history of ankle fractures; a history of foot or ankle deformities, variations, or surgery; and obvious old fractures or osteoarthritis of the ankle found in CT images.

**Figure 1:**
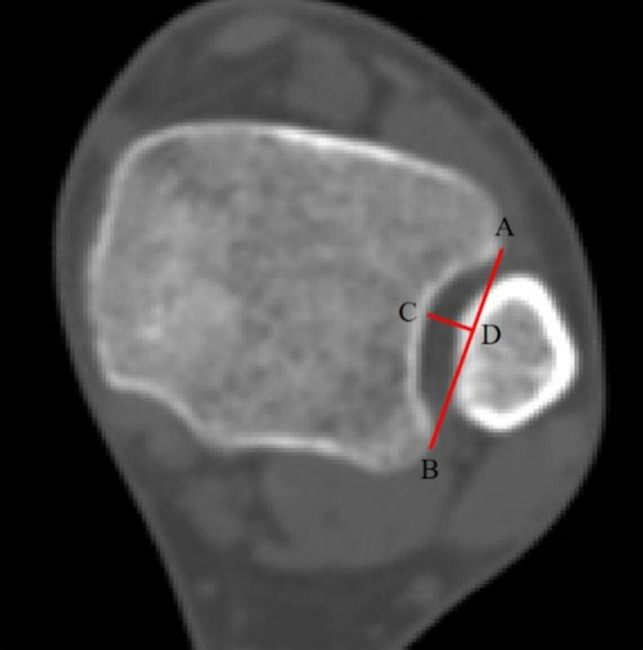
This cross section is located at 1 cm proximal to the distal tibial plafond. Line AB is the tangent line to fibular incisura, and point C is the deepest point of the fibular incisura; through point C, a vertical line is made and intersects line AB at point D, and line CD is the depth of the fibular incisura.

The CT images of normal ankles were imported into MIMICS, and the depth of fibular incisura was measured at the cross section of 1 cm proximal to the distal tibial plafond (**Figure 1**). The depth of fibular incisura ≥4 mm was classified as concave type, and a depth <4 mm was classified as shallow type (20). Then, we reconstructed the 3D models of tibiae, fibulae, and other foot bones with the functions of “segmentation” and “editing.”

**Figure 2:**
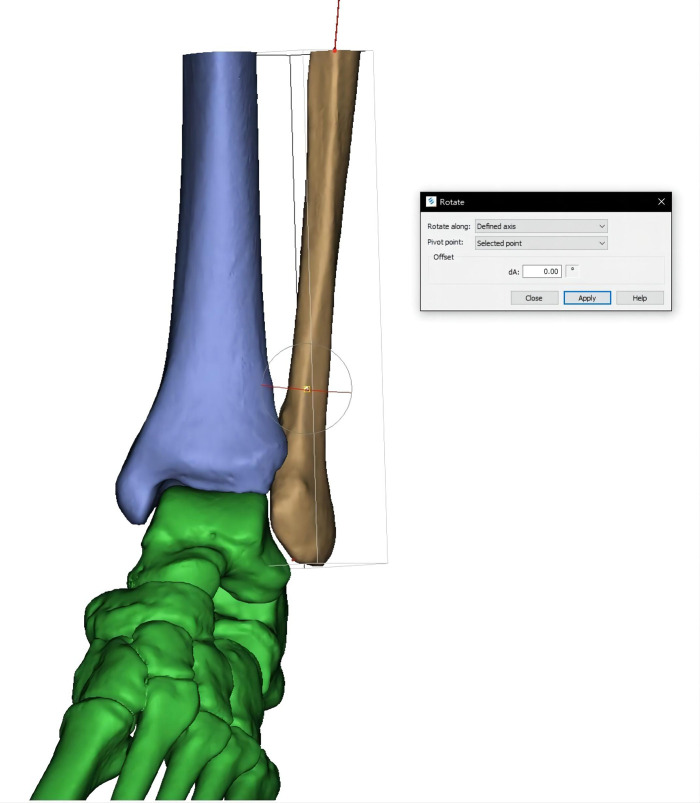
Line coinciding with the center of the fibular medullary cavity is delineated as the longitudinal axis. The fibular model rotates according to the axis to simulate the rotational malreduction.

In a previous cadaveric study, Marmor et al. (18, 21) completely transected the talofibular and syndesmotic ligaments and inserted a 5-mm threaded intramedullary rod into the fibula as a longitudinal axis of rotation. While in our study, ligaments were not reconstructed; we just need to delineate a line that coincides with the center of the fibular medullary cavity as the longitudinal axis (**Figure 2**).

**Figure 3:**
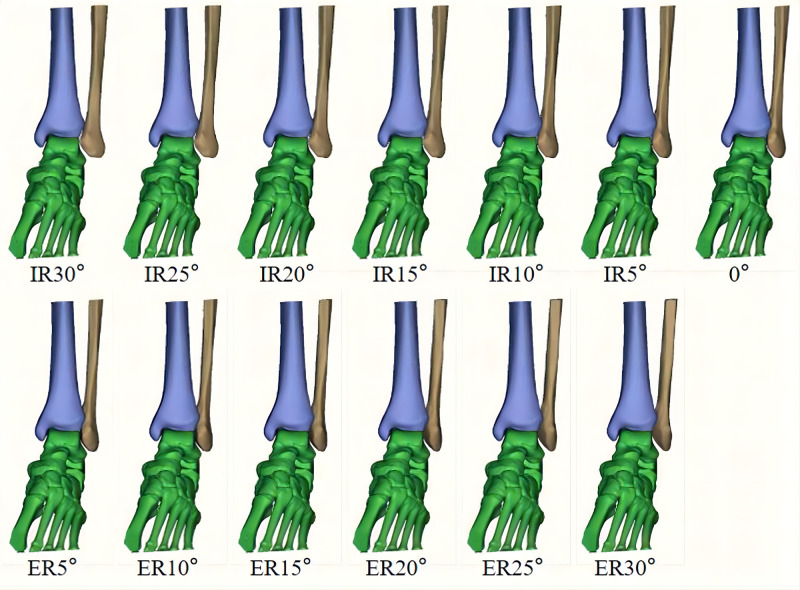
Fibula was rotated both internally and externally along the longitudinal axis to achieve 13 different positions.

The fibula was rotated both internally and externally along the longitudinal axis to achieve 13 distinct positions: neutral (0 degrees); 5 degrees, 10 degrees,15 degrees, 20 degrees, 25 degrees, and 30 degrees of internal rotation; and 5 degrees, 10 degrees, 15 degrees, 20 degrees, 25 degrees, and 30 degrees of external rotation **(Figure 3**). The models of the fibulae may overlap with tibiae or tali after rotation; in reality, this will inevitably lead to lateral movement of the fibulae, so we translated the models of the fibulae laterally along the long axis of the tibiofibular until the models no longer overlapped.

**Figure 4:**
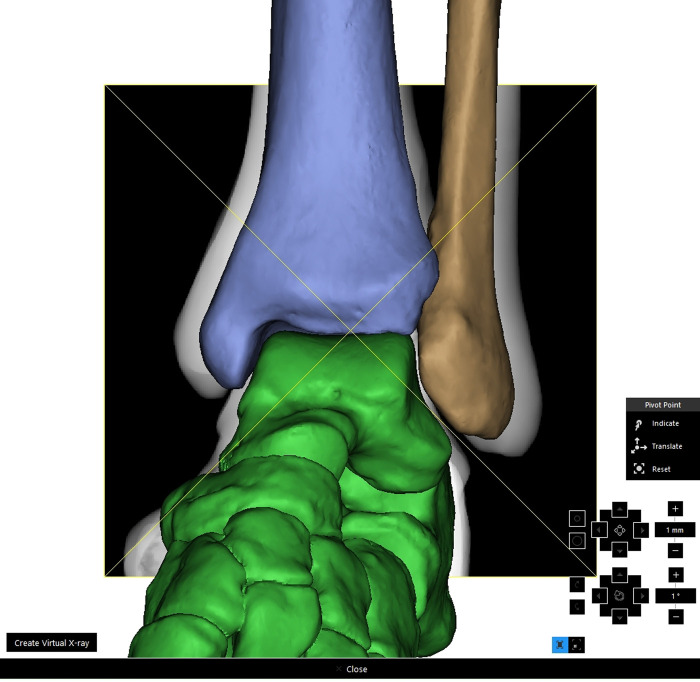
Simulation of intraoperative fluoroscopy with “virtual X-ray” function in MIMICS.

**Figure 5:**
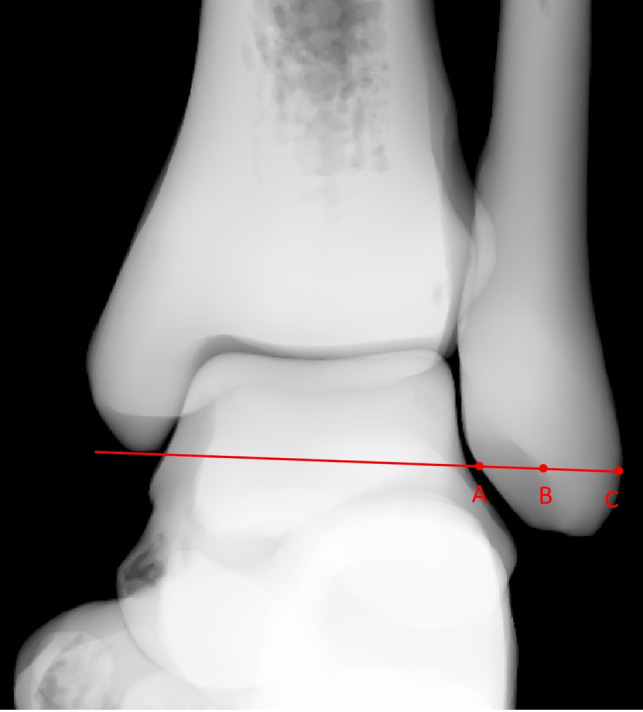
Line drawn through the tip of the medial malleolus and parallel to the distal tibial plafond that intersected the medial edge of the fibula at point A, intersected the lateral malleolar fossa cortex at point B, and intersected the lateral edge of the fibula at point C.

With the virtual X-ray function in MIMICS, total ankle models were internally rotated 15 degrees, and the radiographic images in mortise view were obtained (**Figure 4**). A line was drawn through the tip of the medial malleolus and parallel to the distal tibial plafond; it intersected the medial edge of the fibula at point A, intersected the lateral malleolar fossa cortex at point B, and intersected the lateral edge of the fibula at point C (**Figure 5**). The ratio (ratio *α*) between the lengths of line AB and line AC was calculated. The ratio between the distances of AB and AC was calculated and marked as ratio *α*.

### Statistical Analysis

IBM SPSS statistical software (version 20.0, IBM, Somers, NY, USA) was used for statistical analysis. Two orthopedic surgeons separately measured the ratio α. Intraclass correlation coefficients (ICCs) were calculated to assess interobserver correlations (ICC > 0.70 = acceptable, ICC > 0.80 = accurate). Multiple comparisons were used to compare the differences among the 13 groups of data. Independent-sample *t*-tests were used to compare the differences between the two types of fibular incisura in each group and between the genders in each group. A *p* value <0.05 was considered statistically significant.

## Results

Among the 120 outpatients, 12 were excluded because of previous fracture history, 7 were excluded because they were less than 18 years old, and 5 were excluded due to poor quality of CT images. The remaining 96 were included in the study, with an average age of 38.4 ± 13.6 years, ranging from 18 to 75 years. There were 45 males (46.8%) and 51 females (53.1%). According to the depth of fibular incisura, 40 cases were classified as shallow type, accounting for 41.7%, and 56 cases were classified as concave type, accounting for 58.3%. The mean ratio *α* for the normal ankle was 0.49 ± 0.05 (95% CI, 0.48–0.50) measured by observer 1 and was 0.49 ± 0.06 (95% CI, 0.48–0.50) measured by observer 2; the ICC was 0.96. Further data are listed in **Table 1**. ICCs were all greater than 0.80.

**Table 1:** Mean ± SD, 95% confidence intervals, and ICCs for the observers.

	Observer 1			Observer 2			
		95% Confidence Interval			95% Confidence Interval		
	Mean ± SD	Lower Limit	Uper Limit	Mean ± SD	Lower Limit	Uper Limit	ICC
ER30°	0.27 ± 0.08	0.25	0.29	0.27 ± 0.09	0.25	0.29	0.98
ER25°	0.31 ± 0.08	0.29	0.32	0.31 ± 0.09	0.29	0.33	0.97
ER20°	0.34 ± 0.08	0.33	0.36	0.34 ± 0.09	0.32	0.36	0.97
ER15°	0.38 ± 0.08	0.36	0.39	0.38 ± 0.08	0.36	0.39	0.95
ER10°	0.41 ± 0.07	0.40	0.43	0.42 ± 0.07	0.40	0.43	0.97
ER5°	0.45 ± 0.06	0.44	0.47	0.46 ± 0.07	0.44	0.47	0.96
0°	0.49 ± 0.05	0.48	0.50	0.49 ± 0.06	0.48	0.50	0.96
IR5°	0.52 ± 0.06	0.51	0.53	0.52 ± 0.07	0.51	0.53	0.97
IR10°	0.56 ± 0.06	0.55	0.57	0.56 ± 0.06	0.55	0.57	0.93
IR15°	0.59 ± 0.05	0.58	0.60	0.59 ± 0.07	0.58	0.60	0.93
IR20°	0.61 ± 0.05	0.60	0.62	0.61 ± 0.06	0.60	0.62	0.90
IR25°	0.64 ± 0.05	0.63	0.65	0.64 ± 0.06	0.62	0.65	0.90
IR30°	0.66 ± 0.05	0.65	0.67	0.65 ± 0.07	0.64	0.66	0.87

**Figure 6:**
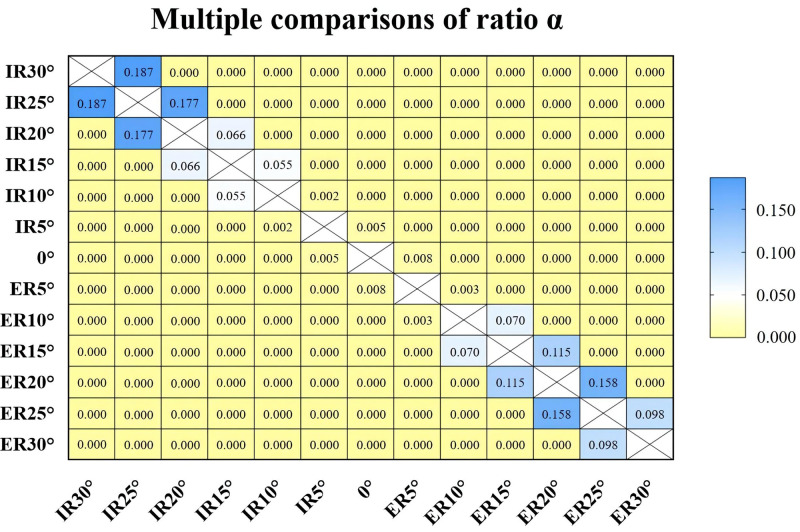
Tamhane’s T2 test was selected according to the data type. The cells with *p* value >0.05 were blue, indicating that there were no statistical significances between the groups. The cells with *p* value <0.05 were yellow, indicating that the statistical differences were significant between groups.

Tamhane’s T2 test was selected according to the type of data. For data measured by observer 1, the *p* values among groups of 10 degrees of ER, 5 degrees of ER, neutral position, 5 degrees of IR, and 10 degrees of IR were all less than 0.05. Among the groups of 15 degrees, 20 degrees, 25 degrees, and 30 degrees of ER or IR, the *p* values were greater than 0.05 only between the adjacent groups (**Figure 6**). Similar results were obtained from the data of observer 2. The *p* values between different incisura types **(Table 2)** or genders **(Table 3)** in each group were all greater than 0.05.

**Table 2:** Effect of different gender on ratio *α*.

	Observer 1			Observer 2		
	Concave type	Shallow type	*P* value	Concave type	Shallow type	*P* value
ER30°	0.26 ± 0.09	0.28 ± 0.08	0.38	0.26 ± 0.09	0.28 ± 0.08	0.26
ER25°	0.30 ± 0.09	0.31 ± 0.07	0.55	0.30 ± 0.09	0.31 ± 0.08	0.56
ER20°	0.34 ± 0.08	0.35 ± 0.08	0.59	0.33 ± 0.09	0.35 ± 0.08	0.45
ER15°	0.38 ± 0.08	0.38 ± 0.07	0.75	0.38 ± 0.08	0.38 ± 0.08	0.84
ER10°	0.41 ± 0.07	0.42 ± 0.07	0.70	0.41 ± 0.08	0.42 ± 0.07	0.54
ER5°	0.45 ± 0.07	0.46 ± 0.06	0.73	0.46 ± 0.08	0.46 ± 0.06	0.88
0°	0.49 ± 0.05	0.48 ± 0.06	0.37	0.49 ± 0.05	0.49 ± 0.06	0.61
IR5°	0.52 ± 0.06	0.52 ± 0.06	0.75	0.52 ± 0.06	0.52 ± 0.06	0.72
IR10°	0.56 ± 0.06	0.56 ± 0.05	0.96	0.55 ± 0.06	0.57 ± 0.07	0.40
IR15°	0.59 ± 0.06	0.59 ± 0.05	0.91	0.58 ± 0.07	0.60 ± 0.06	0.41
IR20°	0.61 ± 0.06	0.62 ± 0.05	0.57	0.61 ± 0.07	0.62 ± 0.06	0.63
IR25°	0.63 ± 0.05	0.64 ± 0.05	0.44	0.63 ± 0.06	0.65 ± 0.05	0.14
IR30°	0.66 ± 0.05	0.66 ± 0.05	0.67	0.64 ± 0.07	0.66 ± 0.06	0.23

**Table 3:** Effect of different types of fibular incisura on ratio *α*.

	Observer 1			Observer 2		
	Male	Female	*P* value	Male	Female	*P* value
ER30°	0.27 ± 0.08	0.27 ± 0.09	0.94	0.27 ± 0.08	0.27 ± 0.09	0.88
ER25°	0.31 ± 0.07	0.31 ± 0.09	0.95	0.31 ± 0.08	0.31 ± 0.09	0.79
ER20°	0.34 ± 0.07	0.34 ± 0.09	1.00	0.34 ± 0.08	0.34 ± 0.09	0.81
ER15°	0.38 ± 0.07	0.38 ± 0.08	0.97	0.38 ± 0.08	0.38 ± 0.08	0.91
ER10°	0.41 ± 0.06	0.42 ± 0.07	0.80	0.42 ± 0.07	0.42 ± 0.08	0.81
ER5°	0.45 ± 0.06	0.46 ± 0.07	0.67	0.45 ± 0.07	0.46 ± 0.08	0.48
0°	0.49 ± 0.06	0.49 ± 0.04	0.89	0.49 ± 0.07	0.49 ± 0.04	0.57
IR5°	0.52 ± 0.07	0.52 ± 0.06	0.67	0.52 ± 0.07	0.52 ± 0.06	0.99
IR10°	0.56 ± 0.06	0.56 ± 0.06	0.81	0.56 ± 0.07	0.56 ± 0.06	0.89
IR15°	0.59 ± 0.06	0.59 ± 0.05	0.83	0.59 ± 0.06	0.59 ± 0.08	0.71
IR20°	0.62 ± 0.05	0.61 ± 0.05	0.57	0.61 ± 0.07	0.61 ± 0.06	0.99
IR25°	0.64 ± 0.05	0.63 ± 0.05	0.50	0.64 ± 0.06	0.64 ± 0.06	0.42
IR30°	0.66 ± 0.05	0.66 ± 0.05	0.41	0.65 ± 0.07	0.65 ± 0.06	0.81

## Discussion

Rotational malreduction of the distal fibula is difficult to detect intraoperatively. Biomechanical research showed that any angle of fibular malrotational will affect the contact pressures of the ankle joint (1, 2), which may lead to chronic ankle instability. Reducing the fibula to the anatomical position accurately is essential to ensure the stability of the talus in the ankle mortise, restore normal ankle biomechanics, and obtain good functional results (22–24).

Rotational malreduction of the distal fibula was considered to be associated with the type of fracture, the location of forceps, and the distal tibiofibular syndesmotic screw (25). However, the lack of highly sensitive intraoperative measurement is another important factor that cannot be ignored. If the malreduction is not detected in time during the operation, it may lead to poor postoperative functional recovery and even the need for a second operation (26). In 2011, Marmor et al. (21) proposed that it is difficult to detect the internal rotation of less than 10 degrees and external rotation of less than 30 degrees of the fibula with conventional fluoroscopy. Such a large angle range may lead to the miss of the fibular rotational malreduction during the operation, which will affect the long-term functional recovery. Therefore, it is necessary to propose a new method that can accurately evaluate the rotational malreduction of the distal fibula intraoperatively. By comparing the morphologic features of the fibula with malreduction at different angles, they proposed that spoon-shaped fibula and/or widening of the TFCs in the mortise view suggested internal rotation of the distal fibula and discontinuity of the Shenton line and/or pointed blade-shaped fibula and/or TFCs narrowing suggested external rotation of the distal fibula (18). Chang et al. (19) added a new parameter to better assess the rotational malreduction of the fibula; when the fibula was not rotated, the lateral wall cortex of the lateral malleolar fossa showed a distinct vertical dense projection in the mortise view, usually located at two-thirds of the malleolar width from medial to lateral. The disappearance of the fossa cortex shadow indicates internal or external rotation of the fibula. These two methods increased the possibility of detecting the malrotation of the distal fibula under conventional fluoroscopy, but they were not able to be quantified, so they can only be roughly evaluated and cannot be accurately correlated with the rotation angle of the fibula. CT has a higher sensitivity for detecting subtle rotational malreduction of the distal fibula than X-ray (27), but radiation hazards, high cost, and inconvenience make it difficult to apply during operation. Using intraoperative 3D fluoroscopy can provide CT-like images, providing an extra perspective than 2D fluoroscopy, and can be used to improve the reduction of the ankle joint (28, 29); however, this method lacks relevant clinical studies, and its effectiveness needs to be verified.

The method proposed in this study was based on the 3D model reconstruction and virtual X-ray function in MIMICS. It utilized normal-ankle CT images, which can be equivalent to cadaver specimens, greatly reducing the difficulty of obtaining samples. The fibula models were rotated to a specific angle by the software for simulating the rotational malreduction; the rotation angle was accurate and controllable, which greatly reduced the human interference in the experiment and had high repeatability. The reference line is a straight line parallel to the plafond and passes through the tip of the medial malleolus on radiographic images, so it will not be affected by the ankle joint space or the position of the talus, but the shortening or separation of the fibula may have possible effects. Because the intersection with the fibula is distal than syndesmosis, it can not only be applied to Weber type C ankle fracture but has significance to Weber type B and part of Weber type A ankle fracture.

The aim of this study was to propose a new method to help evaluate whether the distal fibula has rotational malreduction or not during operation. According to the results, there were statistical differences among groups of 10 degrees of ER, 5 degrees of ER, neutral position, 5 degrees of IR, and 10 degrees of IR. This is because when the rotation angle is smaller, the displacement of the projection of the lateral malleolar fossa cortex corresponding to the same angle interval is larger. On the contrary, when the rotation angle is larger, the displacement of the projection of the lateral malleolar fossa cortex corresponding to the same angle interval is smaller. Therefore, this method is relatively sensitive to subtle rotational malreduction of the distal fibula.

To the best of our knowledge, this is the first method that can quantitatively measure the rotation angle of the fibula on radiographic images. This method corresponds to the ratio *α* to the fibular rotation angle to accurately evaluate the fibular reduction, which can be used both intraoperatively and postoperatively.

This research still has some limitations. First, the resolution of fluoroscopy is not as high as CT, so the evaluation may not be as accurate as that with intraoperative CT scans. Second, measurement during the operation of the ratio *α* requires the assistance of a machine or another person, and visual observation requires rich experience. Third, this is a retrospective study correlated with X-ray and 3D model reconstruction, and clinical results were still lacking. Further studies are needed to verify the clinical feasibility of this method.

## Conclusion

This is a new method to quantitatively evaluate the rotational malreduction of the distal fibula during operation. The ratio *α* can correspond to the angle of fibula rotation. The larger the ratio *α*, the more the internal rotation of the fibula; the smaller the ratio *α*, the more the external rotation of the fibula. Making the ratio *α* close to 0.5 may be an intuitive approach that can be used intraoperatively.

## Data Availability

The original contributions presented in the study are included in the article/Supplementary Material; further inquiries can be directed to the corresponding author/s.
